# Comparative Analysis of Immune Response Genes Induced by a Virulent or Attenuated Strain of *Babesia bigemina*

**DOI:** 10.3390/ijms26020487

**Published:** 2025-01-08

**Authors:** Grecia Martínez-García, Karel Estrada, José J. Lira-Amaya, Rebeca M. Santamaria-Epinosa, María E. Lopez-Arellano, Edda L. Sciutto-Conde, Carmen Rojas-Martinez, Jesus A. Alvarez-Martínez, Alejandro Sánchez-Flores, Julio V. Figueroa-Millán

**Affiliations:** 1 Centro Nacional de Investigación Disciplinaria en Salud Animal e Inocuidad, Instituto Nacional de Investigaciones Forestales, Agrícolas y Pecuarias, Jiutepec 62550, Mexico; martinez.grecia@inifap.gob.mx (G.M.-G.); lira.juan@inifap.gob.mx (J.J.L.-A.); santamaria.rebeca@inifap.gob.mx (R.M.S.-E.); lopez.eugenia@inifap.gob.mx (M.E.L.-A.); rojas.carmen@inifap.gob.mx (C.R.-M.); alvarez.jesus@inifap.gob.mx (J.A.A.-M.); 2 Facultad de Medicina Veterinaria y Zootecnia, Universidad Nacional Autónoma de México, Mexico City 04510, Mexico; 3 Unidad Universitaria de Secuenciación Masiva y Bioinformática, Instituto de Biotecnología, Universidad Nacional Autónoma de México, Cuernavaca 62209, Mexico; karel.estrada@ibt.unam.mx (K.E.); alejandro.sanchez@ibt.unam.mx (A.S.-F.); 4 Instituto de Investigaciones Biomédicas, Universidad Nacional Autónoma de México, Mexico City 04510, Mexico; edda@unam.mx

**Keywords:** *Babesia bigemina*, buffy coat, RNA-seq, differentially expressed genes (DEGs), immune response (IR)

## Abstract

RNA-seq technology has been widely used for the characterization of the transcriptome profile induced by several diseases in both humans and animals. In the present study, RNA-seq was used to identify the differential expression of genes associated with the immune response in cattle infected with two different strains of *Babesia bigemina*, both derived from the same Mexican field isolate, which exhibit distinct phenotypic characteristics: the virulent strain, capable of producing acute clinical signs, and the attenuated strain, capable of stimulating a protective immune response when used as an immunogen with an efficacy greater than 80%. The differential gene expression analysis performed revealed a total of 620 differentially expressed genes (DEGs). However, the intersection of the edgeR and DESeq2 programs used in the bioinformatics analysis only identified 247 DEGs, of which 108 genes were enriched to be closely correlated with the bovine immune response based on gene ontology terms; most of the DEGs obtained encode proteins associated with the major histocompatibility complex, immunoglobulins, and T-cell surface receptors. The infection caused by the attenuated strain induced higher transcription of immune response genes compared to the infection caused by the virulent strain; nonetheless, in both infections, a greater down-regulation than up-regulation was observed. Different immunoglobulin-associated genes were found to be up-regulated in the group inoculated with the attenuated strain, whereas these were down-regulated in the virulent strain-inoculated group. In addition, an up-regulation of the HSPA6, CD163, and SLC11a1 genes was observed in the group inoculated with the virulent strain, previously reported in other Apicomplexan infections. The findings provide relevant information that could contribute to clarifying the immune response associated with an acute bovine babesiosis infection by *B. bigemina*.

## 1. Introduction

Ticks and tick-borne diseases are major health and economic problems affecting livestock production in tropical and subtropical regions of the world [1]. Among tick-borne diseases, babesiosis stands out as the most important arthropod-borne bovine disease and the second-most important vector-borne hemotropic disease in free-living animals [2,3]. Bovine babesiosis is caused by intraerythrocytic protozoans of the genus *Babesia*, with *Babesia bigemina* and *B. bovis* being the most relevant due to their wide distribution, which is intricately linked to the distribution of their vector, the cattle tick of the genus *Rhipicephalus* (*Boophilus*) [2]. The signs of clinical infection include fever, dehydration, anorexia, depression, weakness, lethargy, prostration, and sudden death in some cases. In infections caused by *B. bigemina*, hemolytic anemia and hemoglobinuria are common because of erythrocyte destruction, despite parasitaemias detected by blood smears only affecting 3% of infected erythrocytes [2,4]. Acute disease occurs within the first 7–14 days after protozoan inoculation; meanwhile, a chronic or persistent infection takes place once the disease is resolved, which usually occurs after the first two weeks [5]. Once the *Babesia* parasites are inoculated, the bovine immune response is deployed. Immune cells, such as activated monocytes, neutrophils, and natural killer cells, are important for starting the response. They release antimicrobial agents, secrete cytokines that regulate the inflammatory response, and phagocytize the pathogen [2,6]. Until now, *B. bovis* has mostly served as a study model for understanding the mechanisms of immunity against bovine babesiosis. The release of several cytokines, including interleukin-1beta (IL-1b), 12 (IL-12), 15 (IL-15), 18 (IL-18) and 4 (IL4), tumor necrosis factor-alpha (TNF-α), and interferon α (IFNα) and γ (IFNγ), as well as reactive oxygen species (ROS), such as nitric oxide (NO), has been reported [5,7]. While some studies have been carried out in *B. bigemina* infections, showing the expression of several cytokines such as TNF-α, IFN-γ, IL-6, IL-10, IL-12, and inducible nitric oxide synthase (iNOs), it is imperative to conduct additional investigations to unravel the mechanism by which infected cattle respond to this parasite [8,9].

The severity of the disease can be prevented or reduced by vaccination; cattle immunization is regarded as the most effective approach for the prevention and control of bovine babesiosis [2,10]. In Mexico, a live attenuated strain of *Babesia bigemina* (BIS) was developed. BIS was derived from a parental virulent Mexican field isolate, and virulence loss was achieved by in vitro cultivation adaptation. BIS has been used as an immunogen in several studies under controlled and field conditions, showing an efficacy of at least 80% in vaccinated cattle exposed to virulent strains. However, only the clinical effects and humoral immune response have been evaluated [10,11,12]. It is thus imperative to evaluate the expression of genes associated with the immune response provided by BIS versus that of a virulent *B. bigemina* strain. In order to understand the molecular mechanisms elucidated by both strains on the cattle host and to discern the differences and resemblances among the immune responses evoked, we used RNA-seq technology to assess the responses in this study.

Recent studies have demonstrated the usefulness of next-generation sequencing (NGS), specifically RNA sequencing (RNA-seq), for transcriptomic profile analyses of peripheral blood mononuclear cells from bovines exposed to virulent pathogens and vaccines that protect them against those pathogens [13,14,15,16]. RNA-seq analysis enables the identification of differentially expressed genes (DEGs) among two or more groups of individuals subjected to specific pathological or physiological conditions [17]. Furthermore, the molecular function, biological process, and cellular component of which a gene is part can be predicted through gene ontology analysis [18]. Therefore, RNA-seq allows us to determine the genes that are involved in the molecular pathways associated with the established immune response to pathogen infection. Regarding its use in bovine babesiosis, RNA-seq has been used to characterize the transcriptome induced in the salivary glands of *Rhipicephalus annulatus* infected with *B. bigemina* [19], and to assess gene regulation of *B. bovis* life stages in its vertebrate and tick hosts [20]. Most recently, it has been used in the comparison of the transcriptome exhibited by virulent and attenuated *Babesia bigemina* strains, both derived from the same parasite field isolate [21]. However, until now, no research has been conducted regarding the bovine transcriptome in relation to the immune response induced by a *B. bigemina* or *B. bovis* infection. Under these precedents, the objective of the present study was to analyze the transcriptomic profile related to the immune response involved during the acute phase of babesiosis in cattle inoculated with a virulent or attenuated strain of *Babesia bigemina*, comparing those profiles with the transcriptomic profile of a healthy cattle group and with each other. To accomplish this aim, differentially expressed genes (DEGs) that might be involved in the cattle response to *B. bigemina* were identified by using RNA-seq technology applied to samples of buffy coat cells derived from peripheral blood.

## 2. Results

### 2.1. Sequencing

Paired-end libraries were constructed for each buffy coat sample of the three different experimental groups. Group I (GI) was inoculated with a virulent strain of *B. bigemina*, group II (GII) was inoculated with an attenuated strain of *B. bigemina*, and group III (GIII) was the non-inoculated control group. The number of reads obtained from sequencing performed on NextSeq 500 Illumina^®^ (San Diego CA, USA) ranged from 17,431,266 to 18,832,282 (Table 1). The sequence quality of each sample evaluated with FastQC software (v0.11.5) was good, Phred values ranged from 32 to 36, the GC content was 47 to 55%, and the sequence length was 76 bp.

### 2.2. RNA Sequencing (RNA-Seq) Analysis

**Figure 1:**
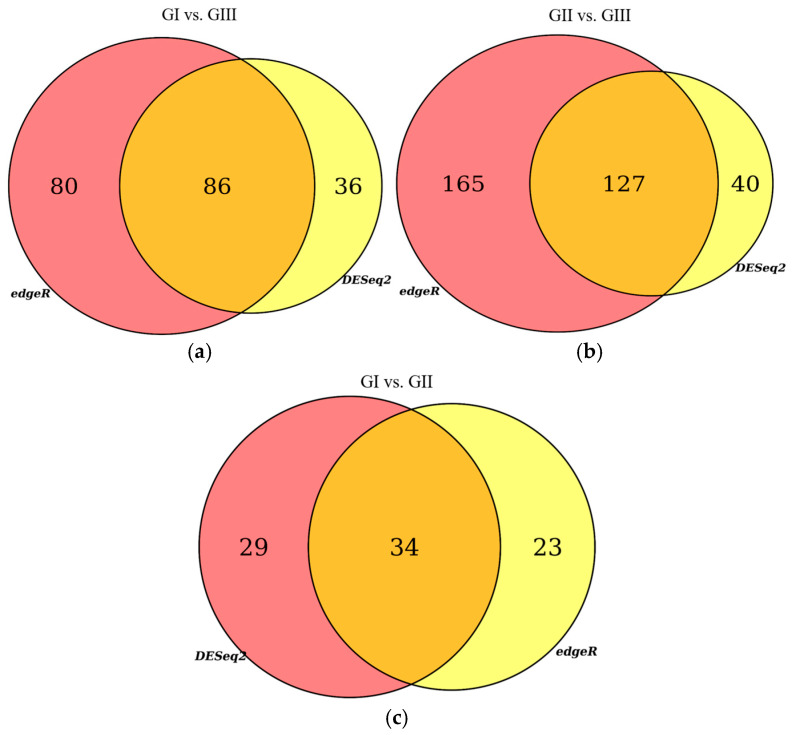
Venn diagrams. Differentially expressed genes (DEGs) identified with edgeR and DESeq2 programs: (**a**) comparison between the group inoculated with a virulent strain of *B. bigemina* (GI) and the non-inoculated control group (GIII); (**b**) comparison between the group inoculated with an attenuated strain of *B. bigemina* (GII) and the non-inoculated control group (GIII); (**c**) comparison between the group inoculated with a virulent strain of *B. bigemina* (GI) and the group inoculated with the attenuated strain of *B. bigemina* (GII).

**Figure 2:**
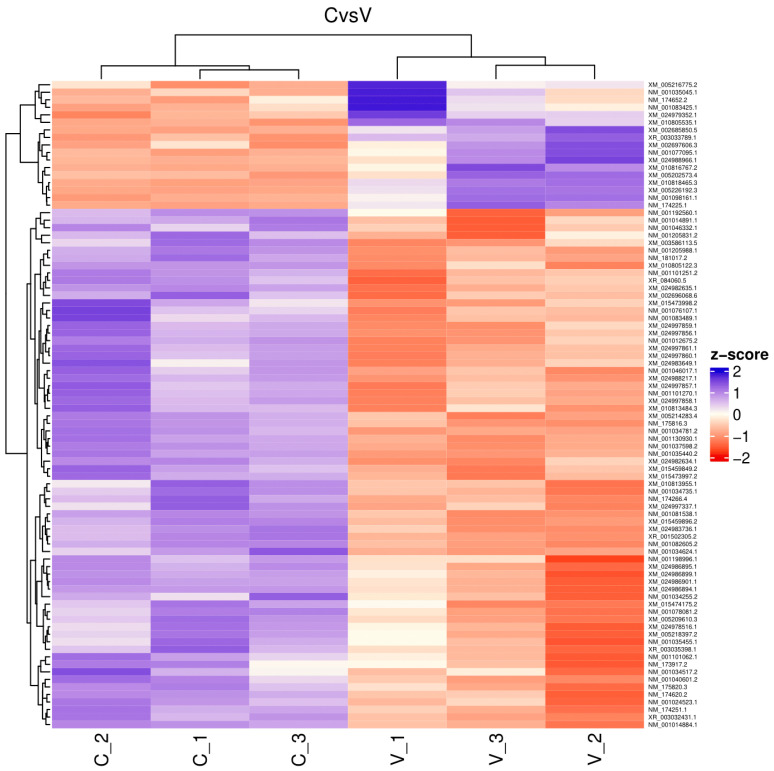
Heatmap. Differentially expressed genes between virulent-strain-inoculated (V_1, V_2, V3) and non-inoculated (C_1, C_2, C_3) bovines. The level of gene expression is represented by colors regarding a Z score: the higher score levels of expression are represented in purple tones, and the lower score levels of expression are represented in red tones.

**Figure 3:**
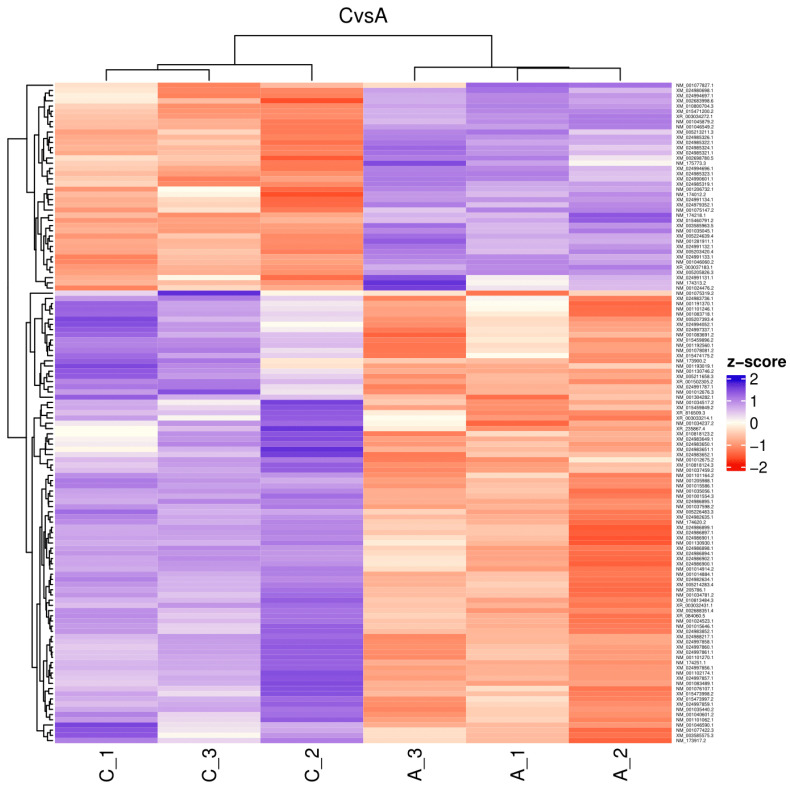
Heatmap. Differentially expressed genes between attenuated-strain-inoculated (A_1, A_2, A3) and non-inoculated (C_1, C_2, C_3) bovines. The level of gene expression is represented by colors regarding a Z score: the higher score levels of expression are represented in purple tones, and the lower score levels of expression are represented in red tones.

**Figure 4:**
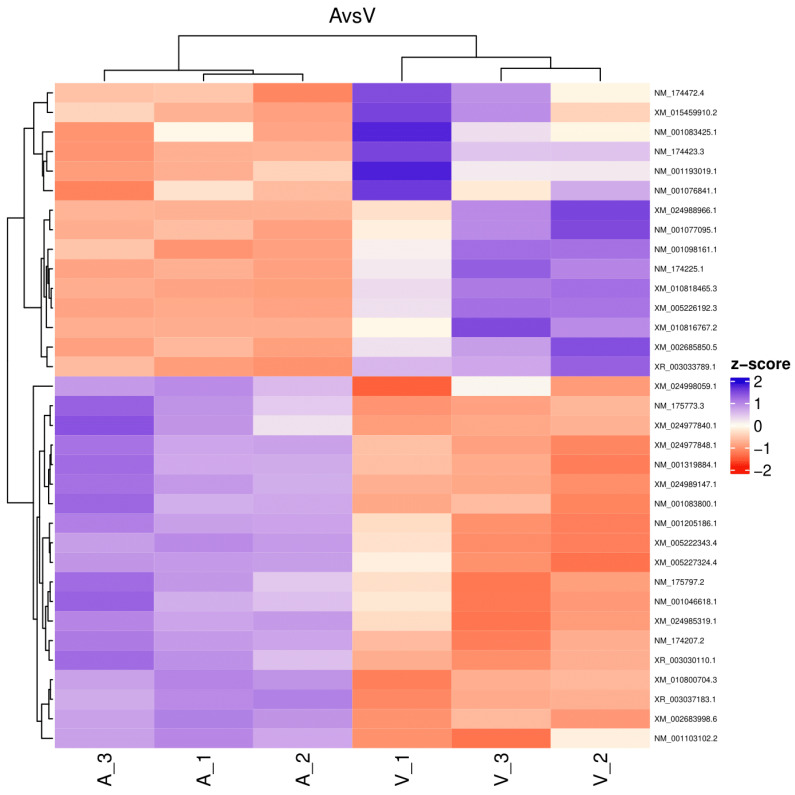
Heatmap. Differentially expressed genes between virulent-strain-inoculated (V_1, V_2, V3) and attenuated-strain-inoculated (A_1, A_2, A3) bovines. The level of gene expression is represented by colors regarding a Z score: the higher score levels of expression are represented in purple tones, and the lower score levels of expression are represented in red tones.

The reads for each sequenced sample were aligned separately to the *Bos taurus* reference genome (GCF_002263795.1). The number of sequences mapped from each sample ranged from 1,118,703 to 15,724,796 (Table 1). A total of 60,094 different transcripts were identified, including messenger RNA (mRNA) and long non-coding RNA (lncRNA). A pairwise comparison was conducted among the three experimental groups for gene expression analysis with the WEB server IDEAMEX, using the edgeR (v3.40.2) and DESeq2 (v1.38.3) programs. To identify the differentially expressed genes, a logFC > 1 and an adjusted *p*-value of 0.05 were used. The comparisons made were GI vs. GIII, GII vs. GIII, and GI vs. GII. A total of 620 differentially expressed genes (DEGs) were identified across the three experimental groups, resulting in the identification of 202, 332, and 86 DEGs between GI vs. GIII, GII vs. GIII, and GI vs. GII, respectively (Figure 1). However, considering the intersection of DESeq2 and edgeR programs, only a total of 247 DEGs were identified and were subsequently used for further analysis, of which 86, 127, and 34 DEGs corresponded to GI vs. GIII, GII vs. GIII, and GI vs. GII comparisons, respectively. The differentially expressed genes found in the intersection between the DESeq2 and edgeR programs from each group paired with each one of the biological replicates are shown in heatmaps (Figure 2, Figure 3 and Figure 4). The gene expression level relative to a Z score can be observed through a color representation; higher scores are depicted in purple tones, while lower scores are depicted in red tones.

The selection of DEGs related to the bovine immune response (BIR) was performed by searching the NCBI platform (https://www.ncbi.nlm.nih.gov/gene, accessed on 27 May 2023). Different characteristics of the differentially expressed transcripts were identified, such as the gene name that corresponds to them, the encoded protein, and especially their possible immune function according to the gene ontology derived from the NCBI Reference Sequence. The gene ontology (GO) terms focus on molecular functions, metabolic processes, and cellular components; in addition, a gene homology search was carried out when the bovine genes did not have a GO description associated with them, with the purpose of linking the genes with their potential immunological function. The DEGs associated with bovine immune responses identified between the comparison of groups GI and GIII are listed in Table 2: a total of 28 genes were detected, with 24 genes down-regulated and 4 genes up-regulated. Among the principal down-regulated genes identified in the virulent strain-inoculated group are the following: RAB27A, a member of the RAS oncogene family (RAB27A); major histocompatibility complex, class II, DQ alpha 5 (BOLA-DQA5); megakaryocyte and platelet inhibitory receptor G6b (MPIG6B); and short transmembrane mitochondrial protein 1 (STMP1). Heat shock protein family A (Hsp70) member 6 (HSPA6), CD163 molecule (CD163), signal transducer and activator of transcription 1 (STAT1), and solute carrier family 11 member 1 (SLC11A1) were the up-regulated genes detected in the above-mentioned group.

The analysis conducted with groups GII and GIII (Table 3) came up with the highest number of DEGs linked to BIR, with 63 DEGs, 35 of which were down-regulated and 28 up-regulated. In this comparison, RAB27A, pro-platelet basic protein (PPBP), CD83 molecule (CD83), and sulfiredoxin 1 (SRXN1) were the main down-regulated genes. Among the up-regulated genes, TCR gamma alternate reading frame protein (TARP), T-cell receptor gamma chain C region C10.5 (LOC100335205), and C-C motif chemokine ligand 4 (CCL4) were found. Finally, the comparative analysis of groups GI and GII is presented in Table 4, with 17 DEGs identified, 13 of which were down-regulated and 4 up-regulated. The main genes that are down-regulated correspond to immunoglobulins: immunoglobulin heavy variable 4-59 (LOC100300806); joining chain of multimeric IgA and IgM (JCHAIN); immunoglobulin heavy variable 4-38-2 (LOC100300716); and Ig heavy chain Mem5-like (LOC100297192). The up-regulated genes included HSPA6; MHC class I heavy chain (LOC507917); NFkB inhibitor delta (NFkBID); and major histocompatibility complex, class I (LOC512672).

In summary, the differential expression analysis performed revealed 108 genes encoding proteins associated with the bovine (*Bos taurus*) humoral immune response against *B. bigemina*; of these, 72 and 36 genes were down- and up-regulated, respectively. The vast majority of these encode proteins of the immunoglobulin (Ig) structure, proteins associated with the major histocompatibility complex (MHC), and T-cell surface receptors. Furthermore, several of the identified transcripts encode the Ras-associated protein, Rab-27A. Although several genes are repeatedly represented, the transcripts to which they belong are different. Therefore, the proteins encoded represent isoforms of the same protein. The relevant features and gene ontology information for every differentially expressed gene can be found in Supplementary Table S1. Additionally, enrichment of GO terms (Webgestalt platform) was performed with the total number of DEGs found by the intersection of the edgeR and DESeq2 programs. This includes all the genes identified by analyzing the cells present in the buffy coat of each experimental group (see Supplementary Table S2).

## 3. Discussion

The next-generation sequencing technology, “RNA-seq”, allows the characterization and analysis of the transcriptional profiles of eukaryotic organisms [22]. RNA-seq results are highly accurate in quantifying gene expression levels compared to real-time PCR (qPCR) testing, and have also demonstrated a high degree of reproducibility between results from biological and technical samples [22,23]. Transcriptomics profiling can be used to compare gene expression between two or more groups of individuals, for example, to analyze samples derived from individuals during the acute phase of a disease, recovered from the disease, or healthy individuals without disease manifestations. Likewise, the information obtained has the potential to be utilized to identify biomarkers useful in the diagnosis and prognosis of diseases [24]. In the present study, the transcriptomic profile expressed by buffy coat cells of peripheral blood derived from bovines experimentally infected with a virulent or attenuated strain of *B. bigemina* was analyzed by RNA-seq. Although several cytokines and molecules that play a role in bovine babesiosis, such as IL-1β, IL-12, IL-15, IL-18, TNF-α, IFNα, IFNγ, and NO, have been identified [5,9], until now, there is insufficient evidence for the immune molecular response induced by *B. bigemina* during the acute phase of the disease or for the gene expression induced by an attenuated *B. bigemina* strain with immunogenic capacity.

The *Bos taurus* genome (GCF_002263795.1) used as a reference for transcript mapping comprises 37,073 genes, of which 21,667 encode proteins and 145 are T-cell receptor and immunoglobulin gene segments, with 63,675 mRNAs and 14,296 ncRNAs (https://www.ncbi.nlm.nih.gov/datasets/genome/GCF_002263795.1/, accessed on 29 May 2023). The data derived from this study revealed 60,094 transcripts, covering 77% of the total transcripts reported in the reference genome. Based upon the analyzed data, 108 out of the 247 DEGs identified by the intersection of DESeq and edgeR programs on the three comparisons performed encode proteins related to the immune response. The principal DEGs identified encode for proteins associated with the major histocompatibility complex, T-cell surface receptors, and immunoglobulins. These findings may be due to the number of days elapsed from inoculation until peripheral blood sampling was performed (samples were taken on 8 days post-inoculation, 8 dpi), when the inflammatory response is predominant [5], with precursor genes for cell differentiation and humoral immune response, as well as genes involved in phagocytosis. The *B. bigemina* virulent-strain-inoculated group showed the highest number of genes sub-expressed, with 24 out of 28 DEGs down-regulated and 4 of 28 DEGs up-regulated. In contrast, 35 out of 63 down-regulated and 28 of 63 up-regulated DEGs were found in the *B. bigemina* attenuated-strain-inoculated group. Similar results have been identified in clinical infections caused by other apicomplexa parasites. For example, in children with various conditions of *Plasmodioum falciparum* infection, widespread down-regulation of gene expression in blood cells has also been reported. The highest number of differentially expressed genes was observed in cerebral malaria as compared to severe malarial anemia, where a considerable number of these DEGs were sub-expressed, which has been proposed as a probable response to the effects of hypoxia [25]. Birds infected with a highly virulent strain of *Plasmodium relictum* have demonstrated a similar trend in gene expression. Over half of the DEGs identified were found to exhibit negative expression 20 dpi, despite the significant overexpression observed after the initial 8 dpi [26]. The gene expression patterns associated with the immune response can change quickly within hours. Vahey et al. [27] reported a significant alteration in the expression of genes associated with the inflammatory response following a third vaccination with the RTS,S subunit recombinant vaccine against malaria. The differential expression of 63 genes was assessed on the vaccine application day as well as 24 and 72 h afterwards. A gene down-regulation profile was observed on vaccination day, but 24 h after the vaccine application, the pattern changed to up-regulation; however, a similar pattern of expression to the vaccination day was observed 72 h later. Hence, the analysis of gene expression in an individual’s transcriptome provides a precise depiction of the genes that are being expressed at the time of sample collection.

The down-regulation of the identified DEGs caused by *B. bigemina* infection may lead to immunosuppression. Previous findings have reported generalized immunosuppression due to other infections caused by Apicomplexan parasites, such as *Plasmodium* spp., *Trypanosoma* spp., and *Leishmania* spp., which may be reflected as a decreased humoral response [28]. Immunosuppression can be attributed to numerous factors, including infection severity, antigenic variation of parasite strains, and high production of non-specific IgM or, conversely, decreased levels of IgM and IgG, as well as mitogens and soluble antigens [29,30]. The comparison of gene expression made between the attenuated and virulent inoculated groups also revealed a greater number of differentially expressed genes, with 13 out of 17 DEGs down-regulated and 4 of 17 up-regulated. Some of the identified genes expressed negatively belong to the immunoglobulin class of proteins, such as the heavy and light chains and the joining chain of multimeric IgA and IgM. Sub-expression of genes was notoriously observed in the virulent *B. bigemina* strain-inoculated group, indicating a probable decrease in the expression of genes associated with the establishment of an adequate humoral response in bovines infected with the virulent strains, as compared to those inoculated with the attenuated *B. bigemina* strain previously used as a live attenuated immunogen.

The RAB27A gene was one of the main transcripts found to be down-regulated in both *B. bigemina*-inoculated groups; it is involved in organelle transport related to lysosomes and secretory granule release. RAB27A has several functions, such as antigen processing and presentation, blood coagulation, complement-dependent cytotoxicity, degranulation of cytotoxic T cells and NK cells, positive regulation of phagocytosis, and positive regulation of the biosynthetic process of reactive oxygen species [31,32]. In eosinophils, it participates in the release of eosinophil peroxidase (EPX) and major basic protein (MBP), crucial molecules for the inflammation process; meanwhile, in neutrophils, it is essential for the activation of the phagocytosis process [33,34]. The decreased expression of various RAB27A isotherms may be linked to elevated activation of T lymphocytes and macrophages, the main characteristic of hemophagocytic syndrome that can develop due to excessive inflammation induced by some infections. RAB27A deficiency is also related to hepatosplenomegaly, neutropenia, and immunodeficiency [34]. The phagocytic activity of neutrophils and monocytes constitutes the initial line of defense against *Babesia* infections, alongside the release of cytokines and chemokines by these cells [5]. The bovine leukocyte antigen (BoLA)-DQA5, another down-regulated gene detected in this study, is expected to participate in various immune processes like those in the human leukocyte antigens (HLA) system. BoLA-DQA5 could be actively participating in the processing and presentation of antigens through MHC class II; in the production of immunoglobulins and the immune response mediated by these molecules; in the positive regulation of T cell activation; as well as in the immune homeostasis maintenance within signaling pathways of the inflammatory and immune response, as well as in various signaling pathways of the mitogen-activated protein kinase (MAPK) cascade related to cellular stress [35,36]. The down-regulation of the BoLA-DQA5 gene in bovine inflammatory diseases such as Epstein–Barr virus infection, inflammatory bowel disease, subclinical endometriosis, and clinical mastitis has been reported. In the last disease, the role of a miRNA called “miR-29c” in the down-regulation of BoLA-DQA5 has been proven. However, it is imperative to conduct additional investigations to ascertain the mechanism of action of this leukocyte antigen [36]. Reduced expression of some homologous genes, such as BOLA-DQA2 and BOLA-DQB, was also identified. The Sulfiredoxin 1 gene was down-regulated in both *B. bigemina*-inoculated groups. The sulfiredoxin-1 (SRXN1) participates in the cellular response to oxidative stress caused by free radicals released, interfering with the correct functioning of cells subjected to stress [37]. Oxidative stress is one of the principal effects caused by Apicomplexas infections. In *Babesia* spp., the release of reactive oxygen species (ROS) and NO is part of the response mechanism for the elimination of parasites [38]. Activated macrophages in the spleen are the cells responsible for the production of babesiacidal molecules such as ROS and nitrogen intermediates, which together with their phagocytic action achieve the elimination of circulating parasites. Other cells capable of releasing NO are monocytes and phagocytic neutrophils [5]. The HSPA6 gene (coding for heat shock protein, Hsp70) was significantly overexpressed in the *B. bigemina* virulent-strain-infected group, which presented the highest increase in rectal temperature during experimental infection. HSPA6 is involved in the cellular response to heat, as well as in the cellular response to unfolded and refolding proteins. In addition, it participates in antigen presentation, stimulates and causes the maturation of antigen-presenting cells, such as macrophages and dendritic cells, and acts as a chaperone for several Toll-like receptors involved in the innate response [39]. The CD163 and STAT1 genes were also up-regulated by the presence of the *B. bigemina* virulent strain. The CD163 molecule is expressed in monocytes and macrophages, participates in the elimination process of hemoglobin/haptoglobin complexes, and has been identified in cattle macrophages infected with *Theileria parva* [40]. In addition, CD163 expression has been reported in *P. falciparum* infections. CD163 is used as a molecular marker associated with parasitemia, and its expression is directly associated with increased parasitemia in infected individuals. It is also likely to be directly linked to lactate levels and inversely to hemoglobin levels [25]. The latter was identified in our study, since the hemoglobin beta gene (HBB) was found to be down-regulated (logFC of −2.27 and *p*-value of 2.08 × 10^−2^) in the same group inoculated with the virulent strain. Conversely, CD163 showed a logFC of 2.76 with a *p*-value of 1.74 × 10^−3^. Free hemoglobin is present during acute infections of *B. bigemina* due to the hemolysis generated by parasite proliferation and the massive destruction of both infected and uninfected erythrocytes by the immune system. The outcome of this disorder could flow into hemolytic anemia and hemoglobinuria, clinical manifestations characteristic of bovine babesiosis [2]. Other cell surface markers such as CD9, CD74, CD79a and CD79b, CD83, and CD247 were also found to be differentially expressed in both *B. bigemina*-infected groups. As for the STAT1 gene, it intervenes in the innate immune response, acting in the positive regulation of the NLRP3 inflammasome complex assembly, which positively regulates the production of the proinflammatory cytokine IL-1β [41]. Monocytes and macrophages produce IL-1β, important for stimulating innate and acquired immunity against tick-borne pathogens [42]. STAT1 has also been identified in fibroblasts exposed to *Toxoplasma gondii*, as a signal transducer acting on a regulator of IFNγ expression [43].

Other DEGs of interest that may be closely related to *Babesia*-induced responses include solute carrier family 11 member 1 (SLC11A1), involved in the defense response to protozoa; platelet factor 4 precursor (PF4), which has demonstrated antimicrobial activity against *P. falciparum*; thioredoxin domain-containing protein 5 precursor (TXNDC5), whose expression is induced by the presence of hypoxia and which probably protects hypoxic cells from apoptosis; and TRAF3-interacting JNK activator modulator isoform 2 (TRAF3IP3), involved in the positive regulation of IFNα production. For instance, SLC11A1, also known as NRAMP1, is expressed by macrophages, monocytes, and lymphocytes. NRAMP1 has multiple effects on macrophage activation, acts as a regulator and transporter of intracellular metals, and is present in lysosomes, endosomes, and phagosomes. It has been reported to be involved during infections caused by intracellular pathogens, such as *Toxoplasma* spp. and *Leishmania* spp.; it is suggested to inhibit the growth of pathogens by limiting the entry of metals into cells [44,45]. The restriction of ion metals like iron and manganese, which are essential for host cells and pathogens, respectively, is known as nutritional immunity. Iron participates in different metabolic pathways, principally in ATP production, while manganese is utilized by pathogens to produce superoxide dismutase, an enzyme necessary to confront the oxidative mechanisms generated by the host to kill the pathogens [45]. Human platelets release the PF4 chemokine upon contact with erythrocytes infected with mature *P. falciparum* parasites, usually infected with the merozoite intraerythrocytic phase. PF4 attaches to parasitized erythrocytes through the atypical chemokine receptor 1, also known as DARC; PF4 is internalized, causing the death of the intracellular parasite. The antimicrobial activity of PF4 has been documented in other *Plasmodium* species, including *P. vivax*, *P. knowlesi*, and *P. chabaudi* [46,47]. Previous studies have revealed the presence of the DARC receptor in the erythrocytes of *Bos taurus* and *Bos indicus* [48], indicating that the administration of PF4 during an infection with *Babesia* spp. may be feasible, although new research is needed to verify whether there is an anti-*Babesia* effect due to the release of PF4 by bovine platelets when in contact with erythrocytes parasitized by *B. bigemina*. Finally, the TXNDC5 gene, also known as ERp46, is associated with cell proliferation and migration, apoptosis, and antioxidant stress [49]. TXNDC5 is overexpressed under hypoxic conditions and autoimmune diseases, functions as a stress survival factor, and is involved in angiogenesis in response to TNF-α and in the inflammatory response through the NF-κB signaling pathway [50]. Though TXNDC5 was up-regulated in the *B. bigemina* attenuated-strain-inoculated group, parasitemia was barely perceptible.

Other highly up-regulated transcripts, with logFC values ranging from 3.12 to 12.03, not associated with the immune response identified in the comparison made between the GI and GII groups, were genes encoding for histones, actins, and tubulin; genes such as H3C10 (logFC 9.63), LOC112445463 (logFC 8.76), and H1-2 (logFC 3.12), as well as ACTA1 (logFC 12.03 and 6.85), ACTBL2 (logFC 7.95), and TUBA8 (logFC 5.13), were identified. This could be the result of the high parasitemia presented in the virulent-strain-inoculated group, promoting greater cell proliferation and mobility.

The enrichment performed through the gene ontology search allowed the identification of differentially expressed genes associated with the immune response, even though some of these genes have only been previously identified and ontologically characterized in the human genome. Although several immune response genes were identified in this study, it cannot be assumed that these were expressed exclusively in response to the infection with *B. bigemina*. Although the experimental cattle were free of infectious diseases before the start of the experiment, and even though the group of uninfected cattle was housed in the same facilities, the development of future experimental research is necessary to verify and/or validate the participation of the reported genes in this study as the exclusive bovine response to a pathogen such as *B. bigemina*. In conclusion, infection with the attenuated strain induces a higher transcription of genes associated with the immune response, compared to the virulent strain infection; however, in both infections, there was a higher up-regulation of genes than down-regulation, being more notable with the virulent strain. Standing out for their function, genes related to immunoglobulin production were up-regulated in the group inoculated with the attenuated strain, while in the group inoculated with the virulent strain, these were down-regulated. Furthermore, an increase in the expression of genes such as HSPA6, CD163, and SLC11a1 was observed in the group inoculated with the virulent strain. These genes have previously been associated with the immune response employed by hosts against other Apicomplexan parasites.

## 4. Materials and Methods

### 4.1. Biological Samples

#### 4.1.1. *Babesia bigemina* Strains

The *B. bigemina* strains used were originally isolated from a clinical case of babesiosis in Mexico. The attenuated strain (BIS) has been used as an immunogen in previous studies to prevent the clinical manifestation of babesiosis, with a demonstrated efficacy of at least 80% [12,51]. Its virulence was decreased by an indefinite number of in vitro culture passages with the Microaerophilic Stationary Phase (MASP) system; since then, it has been alternately maintained in cryopreservation and in vitro culture [51,52,53]. Meanwhile, the virulent strain of *B. bigemina* is maintained in cryopreservation and has been reactivated on several occasions by alternate passages in splenectomized and spleen-intact cattle; its potential to continue being transmitted by vector ticks has also been confirmed [53].

#### 4.1.2. Experimental Animals

Nine *Bos taurus* cattle, over one year old, free of infectious diseases such as brucellosis, tuberculosis, IBR and BDV, were provided from a geographic region free of *R. microplus* ticks. Bovines were confirmed to be free of *B. bigemina* and *B. bovis* by an indirect fluorescent antibody test (IFAT) and nested PCR (nPCR), as previously described [12,54]. The animals were randomly divided into three groups of three bovines: group I (GI) was inoculated with the virulent *B. bigemina* strain, group II (GII) was inoculated with the attenuated *B. bigemina* strain, and group III (GIII) was the non-inoculated control group. The GI and GII groups were inoculated intramuscularly with 1 × 10^8^ erythrocytes infected with *B. bigemina* of each specific strain. Bovines were handled in accordance with the good management practices for the health and welfare of experimental animals at CENID-SAI. The Institutional Subcommittee for the Care and Use of Experimental Animals from the FMVZ-UNAM approved the experimentation protocol carried out (SICUAE.MC-202114-2).

#### 4.1.3. Buffy Coat Cell Isolation

Blood collection from the experimental cattle was carried out on 8 dpi, except in the case of one bovine belonging to group II, which was carried out on 7 dpi, at peak parasitemia. GI bovines had an average rectal temperature of 40 °C with a decrease in their packed cell volume (PCV) value, in addition to presenting parasitized erythrocytes by microscopic examination, with an average parasitemia of ≥8%. Meanwhile, GII presented barely countable parasitemia (≤0.1%), without fever and a minimal decrease in their PCV value. Sampling of each animal was performed by jugular venipuncture with a whole-blood collection bag containing CAD as an anticoagulant solution. Subsequently, the packed cell volume from blood was separated by centrifugation at 450× *g* for 30 min at room temperature: the plasma was removed, while the buffy coat was isolated from the cell pack and stored at −80 °C until use. Blood samples from GIII (non-inoculated group) were collected and handled according to the above procedure.

### 4.2. RNA Extraction

The frozen buffy coat samples were mixed at a 1:10 volume ratio with the RNAlater^®^-ICE Frozen Tissue Transition Solution reagent (Thermo Fisher Scientific, Waltham, MA, USA), following the specifications set by the supplier, and were kept at −20 °C for at least 24 h. Subsequently, the stabilizer was removed, and the total RNA was isolated with the RNeasy^®^ Mini Kit (Qiagen^®^, Hilden, Germany), following the kit protocol provided by the manufacturer. The genomic DNA present in the samples was removed with a DNase I enzyme from the same manufacturer (RNase-Free DNase Set^®^, Hilden, Germany). The concentration and purity of the total RNA isolated from each sample were measured by spectrophotometry with the NP80 equipment (NanoPhotometer^®^ Implen, Munich, Germany). Likewise, the integrity of samples was visualized using the 1% agarose gel electrophoresis technique.

### 4.3. Library Preparation and Sequencing

A sample of total RNA from each bovine with a minimum total concentration of 3 µg was used. cDNA library preparation and RNA-seq sequencing were performed at the Unidad Universitaria de Secuenciación Masiva y Bioinformática (UUSMB) of the Instituto de Biotecnología, UNAM. Library construction was carried out following the TruSeq Stranded mRNA Sample Preparation Kit protocol from Illumina^®^ (San Diego CA, USA). After that, the cDNA libraries were analyzed to determine their average size by a capillary electrophoresis assay using a Bioanalyzer 2100 (Agilent^®^, Santa Clara, CA, USA). Library concentration was determined with the Qubit 1X dsDNA HS Assay Kit (Invitrogen^®^, Thermo Fisher Scientific, Waltham, MA, USA) and by qPCR using Step One equipment (Applied Biosystems^®^, Thermo Fisher Scientific, Waltham, MA, USA). The constructed libraries were normalized to a concentration of 4 nM, and sequencing was performed on NextSeq 500 Illumina^®^ (San Diego CA, USA), generating sequences of 2 × 75 cycles with a total of approximately 10 million reads/sample. The data obtained were demultiplexed and converted into FASTQ files with bcl2fastq2 v2.20 software (Illumina^®^).

### 4.4. RNA-Seq Analysis

The quality control of the sequence reads obtained was performed using FastQC (v0.11.5), and good-quality reads of each sample were mapped to the *B. taurus* reference genomes (GCF_002263795.1) with BWA software (v0.7.17-r1188). The mapped sequences were filtered and sorted, and optical duplicates were marked. The counting and normalization of the mappings were carried out using the Express program (v1.5.1). Differential expression (DE) analysis was conducted with the WEB server “IDEAMEX Integrated Differential Expression Analysis MultiEXperiment” [55], with the edgeR (v3.40.2) and DESeq2 (v1.38.3) programs, and R version 4.2.3. To determine the differentially expressed genes, an adjusted *p*-value < 0.05 and a logarithm of the fold change (logFC) > 1 were used as cut-off values. Finally, a rigorous search on the NCBI platform (https://www.ncbi.nlm.nih.gov/gene) was carried out to determine the characteristics of every differentially expressed gene, with the aim of identifying the genes associated with the bovine immune response. Likewise, an enrichment of GO terms was carried out, with the WEB server “Webgestalt (WEB-based GEne SeT AnaLysis Toolkit)”, considering the intersection of the DEGs predicted by edgeR and DESeq2.

## Figures and Tables

**Table 1:** Summary of the sequencing and FastQC analysis performed. The sequence reads were mapped to the *Bos taurus* cattle reference genome (GCF_002263795.1). Description of the inoculated experimental groups: virulent strain of *B. bigemina* (GI), attenuated strain of *B. bigemina* (GII), and non-inoculated control group (GIII).

Group	Sample ID	Total Number of Reads	Phred Value (Average)	Reads Length (bp)	Mean GC Content (%)	Mapped Sequences
GI	V_1	17,979,636	33	76	51–55	11,426,710
	V_2	18,832,282	34	76	51–55	1,118,703
	V_3	17,928,270	34	76	51–55	2,338,818
GII	A_1	18,097,878	33	76	51–55	15,724,796
	A_2	17,894,634	33	76	51–55	15,225,955
	A_3	18,148,942	33	76	47–51	14,490,409
GIII	C_1	17,979,610	33	76	47–51	13,518,261
	C_2	18,407,034	33	76	47–51	13,342,950
	C_3	17,431,266	33	76	51–55	14,263,462

**Table 2:** Differentially expressed genes associated with immune response. Comparison between the cattle inoculated with a virulent strain of *B. bigemina* (GI) and non-inoculated (GIII) cattle.

	Transcript	logFC	*p*-Value	Gen Symbol	Gen Name	Encoded Protein	**Protein Name**
Down- regulated	XM_024997861.1	−4.95	2.62 × 10^−9^	RAB27A	RAB27A, member of RAS oncogene family	XP_024853629.1	ras-related protein Rab-27A isoform X3
	XM_024997858.1	−4.73	5.32 × 10^−9^	RAB27A	RAB27A, member of RAS oncogene family	XP_024853626.1	ras-related protein Rab-27A isoform X1
	XM_024997860.1	−4.71	7.80 × 10^−9^	RAB27A	RAB27A, member of RAS oncogene family	XP_024853628.1	ras-related protein Rab-27A isoform X1
	XM_024997856.1	−4.48	2.27 × 10^−8^	RAB27A	RAB27A, member of RAS oncogene family	XP_024853624.1	ras-related protein Rab-27A isoform X3
	NM_001101270.1	−4.33	6.27 × 10^−8^	RAB27A	RAB27A, member of RAS oncogene family	NP_001094740.1	ras-related protein Rab-27A
	XM_024997857.1	−4.22	1.55 × 10^−7^	RAB27A	RAB27A, member of RAS oncogene family	XP_024853624.1	ras-related protein Rab-27A isoform X3
	XM_024997859.1	−4.17	2.22 × 10^−7^	RAB27A	RAB27A, member of RAS oncogene family	XP_024853627.1	ras-related protein Rab-27A isoform X3
	NM_001012675.2	−4.13	8.77 × 10^−4^	BOLA-DQA5	major histocompatibility complex, class II, DQ alpha 5	NP_001012693.2	major histocompatibility complex, class II, DQ alpha 5 precursor
	XM_024983736.1	−3.19	3.47 × 10^−5^	MPIG6B	megakaryocyte and platelet inhibitory receptor G6b	XP_024839504.1	protein G6b isoform X1
	XM_015459849.2	−2.96	4.91 × 10^−3^	BOLA-DQA5	major histocompatibility complex, class II, DQ alpha 5	XP_015315335.1	major histocompatibility complex, class II, DQ alpha 5 isoform X1
	XM_015459896.2	−2.89	3.32 × 10^−4^	MPIG6B	megakaryocyte and platelet inhibitory receptor G6b	XP_015315382.2	protein G6b isoform X2
	NM_001078081.2	−2.85	4.78 × 10^−4^	MPIG6B	megakaryocyte and platelet inhibitory receptor G6b	NP_001071549.1	megakaryocyte and platelet inhibitory receptor G6b precursor
	NM_001130930.1	−2.82	5.84 × 10^−4^	STMP1	short transmembrane mitochondrial protein 1	NP_001124402.1	short transmembrane mitochondrial protein 1
	XM_024983649.1	−2.77	1.17 × 10^−2^	MAPK14	mitogen-activated protein kinase 14	XP_024839417.1	mitogen-activated protein kinase 14 isoform X3
	XM_002696068.6	−2.75	1.75 × 10^−2^	CD79B	CD79b molecule	XP_002696114.1	B-cell antigen receptor complex-associated protein beta chain isoform X2
	NM_001101062.1	−2.71	2.37 × 10^−3^	PF4	platelet factor 4	NP_001094532.1	platelet factor 4 precursor
	NM_001101251.2	−2.69	2.66 × 10^−2^	IFI30	IFI30 lysosomal thiol reductase	NP_001094721.1	gamma-interferon-inducible lysosomal thiol reductase precursor
	NM_001205988.1	−2.67	1.99 × 10^−3^	SRXN1	sulfiredoxin 1	NP_001192917.1	sulfiredoxin-1
	NM_174266.4	−2.6	2.17 × 10^−3^	CD79A	CD79a molecule	NP_776691.2	B-cell antigen receptor complex-associated protein alpha chain precursor
	NM_001034735.1	−2.5	5.50 × 10^−3^	CD74	CD74 molecule	NP_001029907.1	HLA class II histocompatibility antigen gamma chain
	XM_005209610.3	−2.26	1.59 × 10^−2^	CD74	CD74 molecule	XP_005209667.1	HLA class II histocompatibility antigen gamma chain isoform X1
	NM_001192560.1	−2.22	1.33 × 10^−2^	GRAP2	GRB2-related adaptor protein 2	NP_001179489.1	GRB2-related adapter protein 2
	NM_001034517.2	−2.2	2.36 × 10^−2^	LAMTOR5	late endosomal/lysosomal adaptor, MAPK and MTOR activator 5	NP_001029689.1	regulator complex protein LAMTOR5
	NM_001034255.2	−2.18	1.72 × 10^−2^	CFD	complement factor D	NP_001029427.1	complement factor D precursor
Up- regulated	XM_002685850.5	8.35	5.67 × 10^−8^	HSPA6	heat shock protein family A (Hsp70) member 6	XP_002685896.1	heat shock 70 kDa protein 6
	XM_010805535.1	2.76	1.74 × 10^−3^	CD163	CD163 molecule	XP_010803837.1	scavenger receptor cysteine-rich type 1 protein M130 isoform X1
	XM_005202573.4	2.56	1.11 × 10^−2^	STAT1	signal transducer and activator of transcription 1	XP_005202630.1	signal transducer and activator of transcription 1-alpha/beta
	NM_174652.2	2.17	3.11 × 10^−2^	SLC11A1	solute carrier family 11 member 1	NP_777077.1	natural resistance-associated macrophage protein 1

**Table 3:** Differentially expressed genes associated with immune response. Comparison between the cattle inoculated with an attenuated strain of *B. bigemina* (GII) and non-inoculated (GIII) cattle.

	Transcript	logFC	*p*-Value	Gen Symbol	Gen Name	Encoded Protein	Protein Name
Down- regulated	XM_024997856.1	−4.73	1.14 × 10^−11^	RAB27A	RAB27A, member of RAS oncogene family	XP_024853624.1	ras-related protein Rab-27A isoform X3
	XM_024997857.1	−4.4	7.16 × 10^−10^	RAB27A	RAB27A, member of RAS oncogene family	XP_024853624.1	ras-related protein Rab-27A isoform X3
	XM_024997861.1	−4.19	4.41 × 10^−9^	RAB27A	RAB27A, member of RAS oncogene family	XP_024853629.1	ras-related protein Rab-27A isoform X3
	NM_001101270.1	−4.16	7.78 × 10^−9^	RAB27A	RAB27A, member of RAS oncogene family	NP_001094740.1	ras-related protein Rab-27A
	XM_024997858.1	−3.91	3.36 × 10^−9^	RAB27A	RAB27A, member of RAS oncogene family	XP_024853626.1	ras-related protein Rab-27A isoform X1
	XM_024997859.1	−3.88	1.59 × 10^−4^	RAB27A	RAB27A, member of RAS oncogene family	XP_024853627.1	ras-related protein Rab-27A isoform X3
	XM_024997860.1	−3.74	3.57 × 10^−8^	RAB27A	RAB27A, member of RAS oncogene family	XP_024853628.1	ras-related protein Rab-27A isoform X1
	XM_002688351.4	−3.22	1.36 × 10^−5^	PPBP	pro-platelet basic protein	XP_002688397.1	platelet basic protein
	NM_001046590.1	−3.16	1.46 × 10^−4^	CD83	CD83 molecule	NP_001040055.1	CD83 antigen precursor
	NM_001205988.1	−3.11	2.08 × 10^−7^	SRXN1	sulfiredoxin 1	NP_001192917.1	sulfiredoxin-1
	XM_010818124.3	−2.72	1.32 × 10^−4^	MAPK14	mitogen-activated protein kinase 14	XP_010816426.1	mitogen-activated protein kinase 14 isoform X4
	NM_001101062.1	−2.66	2.41 × 10^−4^	PF4	platelet factor 4	NP_001094532.1	platelet factor 4 precursor
	XM_024983649.1	−2.62	5.35 × 10^−4^	MAPK14	mitogen-activated protein kinase 14	XP_024839417.1	mitogen-activated protein kinase 14 isoform X3
	XM_024983652.1	−2.53	2.26 × 10^−3^	MAPK14	mitogen-activated protein kinase 14	XP_024839420.1	mitogen-activated protein kinase 14 isoform X7
	NM_001034517.2	−2.52	3.73 × 10^−4^	LAMTOR5	late endosomal/lysosomal adaptor, MAPK and MTOR activator 5	NP_001029689.1	regulator complex protein LAMTOR5
	NM_001078081.2	−2.52	3.90 × 10^−4^	MPIG6B	megakaryocyte and platelet inhibitory receptor G6b	NP_001071549.1	megakaryocyte and platelet inhibitory receptor G6b precursor
	XM_024991787.1	−2.47	1.27 × 10^−3^	GRAP2	GRB2 related adaptor protein 2	XP_024847555.1	GRB2-related adapter protein 2 isoform X1
	NM_001130930.1	−2.33	1.84 × 10^−3^	STMP1	short transmembrane mitochondrial protein 1	NP_001124402.1	short transmembrane mitochondrial protein 1
	NM_001102174.1	−2.3	2.09 × 10^−3^	MAPK14	mitogen-activated protein kinase 14	NP_001095644.1	mitogen-activated protein kinase 14
	NM_173900.2	−2.29	6.37 × 10^−3^	CD9	CD9 molecule	NP_776325.1	CD9 antigen
	NM_001192560.1	−2.28	4.60 × 10^−4^	GRAP2	GRB2-related adaptor protein 2	NP_001179489.1	GRB2-related adapter protein 2
	XM_015459896.2	−2.26	1.01 × 10^−3^	MPIG6B	megakaryocyte and platelet inhibitory receptor G6b	XP_015315382.2	protein G6b isoform X2
	XM_024983736.1	−2.26	8.38 × 10^−3^	MPIG6B	megakaryocyte and platelet inhibitory receptor G6b	XP_024839504.1	protein G6b isoform X1
	XM_024983650.1	−2.24	3.52 × 10^−3^	MAPK14	mitogen-activated protein kinase 14	XP_024839418.1	mitogen-activated protein kinase 14 isoform X4
	NM_001012676.3	−2.22	1.66 × 10^−3^	BOLA-DQB	major histocompatibility complex, class II, DQ beta	NP_001012694.2	major histocompatibility complex, class II, DQ beta precursor
	NM_001012675.2	−2.18	3.42 × 10^−3^	BOLA-DQA5	major histocompatibility complex, class II, DQ alpha 5	NP_001012693.2	major histocompatibility complex, class II, DQ alpha 5 precursor
	XM_010818123.2	−2.15	5.92 × 10^−3^	MAPK14	mitogen-activated protein kinase 14	XP_010816425.1	mitogen-activated protein kinase 14 isoform X1
	XM_024983651.1	−2.1	1.18 × 10^−2^	MAPK14	mitogen-activated protein kinase 14	XP_024839419.1	mitogen-activated protein kinase 14 isoform X5
	NM_001101246.1	−2.09	1.01 × 10^−2^	MTURN	maturin, neural progenitor differentiation regulator homolog	NP_001094716.1	maturin
	XM_024983852.1	−2.08	1.50 × 10^−3^	BOLA-DQA2	major histocompatibility complex, class II, DQ alpha 2	XP_024839620.1	major histocompatibility complex, class II, DQ alpha 2
	NM_001193019.1	−1.98	2.17 × 10^−2^	NFKBID	NFKB inhibitor delta	NP_001179948.1	NF-kappa-B inhibitor delta
	NM_001130746.2	−1.94	9.97 × 10^−3^	NFKBIE	NFKB inhibitor epsilon	NP_001124218.1	NF-kappa-B inhibitor epsilon
	XM_015459849.2	−1.9	1.10 × 10^−2^	BOLA-DQA5	major histocompatibility complex, class II, DQ alpha 5	XP_015315335.1	major histocompatibility complex, class II, DQ alpha 5 isoform X1
	XM_005207393.4	−1.82	2.75 × 10^−2^	GRAP2	GRB2-related adaptor protein 2	XP_005207450.1	GRB2-related adapter protein 2 isoform X1
	NM_001101164.2	−1.78	1.04 × 10^−2^	EGLN3	egl-9 family hypoxia-inducible factor 3	NP_001094634.1	prolyl hydroxylase EGLN3
Up- regulated	XM_003585963.5	3.5	4.42 × 10^−7^	TARP	TCR gamma alternate reading frame protein	XP_003586011.2	uncharacterized protein LOC100335800 isoform X1
	XM_024991132.1	3.06	1.34 × 10^−6^	LOC100335205	T-cell receptor gamma chain C region C10.5	XP_024846900.1	T-cell receptor gamma chain C region C10.5 isoform X2
	NM_001075147.2	2.59	9.94 × 10^−4^	CCL4	C-C motif chemokine ligand 4	NP_001068615.1	C-C motif chemokine 4 precursor
	XM_024985324.1	2.59	1.09 × 10^−4^	LOC618541	uncharacterized LOC618541	XP_024841092.1	uncharacterized protein LOC618541 isoform X1
	NM_001281911.1	2.55	3.34 × 10^−4^	TARP	TCR gamma alternate reading frame protein	NP_001268840.1	uncharacterized protein LOC100335800
	XM_024985322.1	2.44	3.85 × 10^−4^	LOC618541	uncharacterized LOC618541	XP_024841090.1	uncharacterized protein LOC618541 isoform X1
	XM_024991133.1	2.37	1.52 × 10^−4^	LOC100335205	T-cell receptor gamma chain C region C10.5	XP_024846901.1	T-cell receptor gamma chain C region C10.5 isoform X3
	NM_001046060.2	2.25	1.65 × 10^−3^	GIMAP4	GTPase, IMAP family member 4	NP_001039525.1	GTPase IMAP family member 4
	XM_024985326.1	2.25	7.58 × 10^−4^	LOC618541	uncharacterized LOC618541	XP_024841094.1	uncharacterized protein LOC618541 isoform X2
	XM_024985321.1	2.23	1.03 × 10^−3^	LOC618541	uncharacterized LOC618541	XP_024841089.1	uncharacterized protein LOC618541 isoform X1
	XM_010800704.3	2.21	3.44 × 10^−4^	LOC100300806	immunoglobulin heavy variable 4-59	XP_010799006.3	immunoglobulin heavy variable 4-59 isoform X1
	XM_024994697.1	2.17	3.73 × 10^−3^	TCF7	transcription factor 7	XP_024850465.1	transcription factor 7 isoform X5
	XM_024985323.1	2.16	9.03 × 10^−4^	LOC618541	uncharacterized LOC618541	XP_024841091.1	uncharacterized protein LOC618541 isoform X1
	XM_024985319.1	2.09	1.03 × 10^−3^	IL32	interleukin 32	XP_024841087.1	uncharacterized protein IL32 isoform X3
	XM_005203420.4	2.06	9.33 × 10^−4^	CD247	CD247 molecule	XP_005203477.1	T-cell surface glycoprotein CD3 zeta chain isoform X1
	NM_174012.2	2.05	1.10 × 10^−2^	CD247	CD247 molecule	NP_776437.1	T-cell surface glycoprotein CD3 zeta chain precursor
	XM_005224639.4	2.03	1.43 × 10^−3^	IL32	interleukin 32	XP_005224696.1	interleukin-32 isoform X1
	XM_024990601.1	1.97	6.77 × 10^−3^	GIMAP4	GTPase, IMAP family member 4	XP_024846369.1	GTPase IMAP family member 4 isoform X1
	NM_175773.3	1.95	1.04 × 10^−2^	JCHAIN	joining chain of multimeric IgA and IgM	NP_786967.1	immunoglobulin J chain precursor
	XM_005205826.3	1.94	5.01 × 10^−3^	GIMAP4	GTPase, IMAP family member 4	XP_005205883.1	GTPase IMAP family member 4 isoform X1
	NM_001024476.2	1.91	1.89 × 10^−2^	TRAF3IP3	TRAF3-interacting protein 3	NP_001019647.2	TRAF3-interacting JNK-activating modulator isoform 2
	XM_024991131.1	1.88	3.02 × 10^−2^	LOC100335205	T-cell receptor gamma chain C region C10.5	XP_024846899.1	T-cell receptor gamma chain C region C10.5
	XM_015471200.2	1.86	3.26 × 10^−3^	IL2RB	interleukin 2 receptor subunit beta	XP_015326686.2	interleukin-2 receptor subunit beta
	XM_002683998.6	1.85	1.73 × 10^−2^	LOC100300716	immunoglobulin heavy variable 4-38-2	XP_002684044.5	immunoglobulin heavy variable 4-38-2
	XM_024991134.1	1.85	7.11 × 10^−3^	LOC100335205	T-cell receptor gamma chain C region C10.5	XP_024846902.1	T-cell receptor gamma chain C region C10.5 isoform X4
	XM_024994696.1	1.75	1.69 × 10^−2^	TCF7	transcription factor 7	XP_024850464.1	transcription factor 7 isoform X3
	NM_001046549.2	1.72	8.58 × 10^−3^	TUBB	tubulin beta class I	NP_001040014.1	tubulin beta-5 chain
	NM_001206732.1	1.71	2.92 × 10^−2^	TXNDC5	thioredoxin domain-containing 5	NP_001193661.1	thioredoxin domain-containing protein 5 precursor

**Table 4:** Differentially expressed genes associated with immune response. Comparison between the cattle inoculated with a virulent strain of *B. bigemina* (GI) and attenuated strain of *B. bigemina* (GII).

	Transcript	logFC	*p*-Value	Gen Symbol	Gen Name	Encoded Protein	Protein Name
Down- regulated	XM_010800704.3	−3.65	3.42 × 10^−5^	LOC100300806	immunoglobulin heavy variable 4-59	XP_010799006.3	immunoglobulin heavy variable 4-59 isoform X1
	NM_175773.3	−3.49	5.92 × 10^−5^	JCHAIN	joining chain of multimeric IgA and IgM	NP_786967.1	immunoglobulin J chain precursor
	XM_002683998.6	−3.48	5.85 × 10^−5^	LOC100300716	immunoglobulin heavy variable 4-38-2	XP_002684044.5	immunoglobulin heavy variable 4-38-2
	NM_001319884.1	−3.4	1.58 × 10^−4^	LOC100297192	Ig heavy chain Mem5-like	NP_001306813	Ig heavy chain Mem5-like precursor
	XM_024989147.1	−3.35	2.19 × 10^−4^	LOC112441460	immunoglobulin lambda-1 light chain	XP_024844915.1	immunoglobulin lambda-1 light chain
	XM_024977848.1	−3.32	2.77 × 10^−4^	LOC112441499	immunoglobulin lambda-1 light chain	XP_024833616.2	immunoglobulin lambda-1 light chain
	XM_005222343.4	−3.17	2.52 × 10^−4^	LOC104968484	immunoglobulin heavy variable 4-59	XP_005222400.3	immunoglobulin heavy variable 4-59
	NM_001083800.1	−2.92	2.72 × 10^−3^	LOC789205	immunoglobulin lambda-1 light chain-like	NP_001077269.1	immunoglobulin lambda-1 light chain-like precursor
	NM_001103102.2	−2.86	3.57 × 10^−3^	SPN	Sialophorin	NP_001096572	Leukosialin
	NM_001046618.1	−2.7	4.41 × 10^−3^	PTPRCAP	protein tyrosine phosphatase receptor type C-associated protein	NP_001040083.1	protein tyrosine phosphatase receptor type C-associated protein precursor
	NM_001205186.1	−2.57	1.86 × 10^−2^	LOC524810	IgM	NP_001192115.1	IgM precursor
	XM_005227324.4	−2.32	1.87 × 10^−2^	LSP1	lymphocyte-specific protein 1	XP_005227381.1	lymphocyte-specific protein 1 isoform X1
	XM_024985319.1	−2.22	2.95 × 10^−2^	IL32	interleukin 32	XP_024841087.1	uncharacterized protein IL32 isoform X3
Up- regulated	XM_002685850.5	8.4	3.90 × 10^−4^	HSPA6	heat shock protein family A (Hsp70) member 6	XP_002685896.1	heat shock 70 kDa protein 6
	XM_015459910.2	3.76	2.61 × 10^−3^	LOC507917	MHC class I heavy chain	XP_015315396.1	BOLA class I histocompatibility antigen, alpha chain BL3-6 isoform X1
	NM_001193019.1	2.48	2.13 × 10^−2^	NFKBID	NFKB inhibitor delta	NP_001179948.1	NF-kappa-B inhibitor delta
	NM_001076841.1	2.3	3.41 × 10^−2^	LOC512672	major histocompatibility complex, class I	NP_001070309.1	major histocompatibility complex, class I precursor

## Data Availability

Data are contained within the article and Supplementary Materials. Nucleotide sequence data reported in this paper are available in the NCBI SRA database under the BioProject accession number PRJNA1185254 (https://www.ncbi.nlm.nih.gov/sra/PRJNA1185254, accessed on 29 May 2023).

## Supplementary Materials

The following supporting information can be downloaded at https://www.mdpi.com/article/10.3390/ijms26020487/s1.
